# The influence of a hydraulic prosthetic ankle on residual limb loading during sloped walking

**DOI:** 10.1371/journal.pone.0173423

**Published:** 2017-03-09

**Authors:** Sara R. Koehler-McNicholas, Eric A. Nickel, Joseph Medvec, Kyle Barrons, Spencer Mion, Andrew H. Hansen

**Affiliations:** 1 Minneapolis Department of Veterans Affairs Health Care System, Minneapolis, Minnesota, United States of America; 2 Program in Rehabilitation Science, Department of Rehabilitation Medicine, University of Minnesota, Minneapolis, Minnesota, United States of America; University of Colorado Boulder, UNITED STATES

## Abstract

In recent years, numerous prosthetic ankle-foot devices have been developed to address the demands of sloped walking for individuals with lower-limb amputation. The goal of this study was to compare the performance of a passive, hydraulic ankle-foot prosthesis to two related, non-hydraulic ankles based on their ability to minimize the socket reaction moments of individuals with transtibial amputation during a range of sloped walking tasks. After a two-week accommodation period, kinematic data were collected on seven subjects with a transtibial amputation walking on an instrumented treadmill set at various slopes. Overall, this study was unable to find significant differences in the torque at the distal end of the prosthetic socket between an ankle-foot prosthesis with a hydraulic range-of-motion and other related ankle-foot prosthesis designs (rigid ankle, multiaxial ankle) during the single-support phase of walking. In addition, socket comfort and perceived exertion were not significantly different for any of the ankle-foot prostheses tested in this study. These results suggest the need for further work to determine if more advanced designs (e.g., those with microprocessor control of hydraulic features, powered ankle-foot designs) can provide more biomimetic function to prosthesis users.

## Introduction

The ability to walk on sloped terrain facilitates many aspects of community participation (e.g., negotiating ramps, curb cutouts, and naturally-occurring topographical features). Studies show that in order to walk on sloped terrain, able-bodied individuals adjust the posture of their lower limbs, exhibiting significant changes in ankle and knee motion compared to walking on level surfaces [1,2]. For individuals with transtibial amputation, the ability to walk on sloped terrain is significantly compromised due to lost anatomy and altered function of muscles proximal to the amputation. Although commercially-available ankle-foot prostheses restore some of this function, most ankle-foot prostheses do not allow true ankle motion. For example, prosthetic ankle-foot devices that incorporate rigid ankles generally attempt to replace the actions of the biologic ankle-foot system through deformations of their materials. Alternatively, prosthetic ankle-foot devices that do incorporate ankle motion usually allow rotational motion about one equilibrium angle that only changes with mechanical adjustments (e.g., alignment) of the prosthesis [3]. Some of these devices use springs and/or bumpers to store and release energy and return the device’s ankle joint to its equilibrium angle. This approach can result in good function on level terrain when using shoes of one particular heel height. However, problems can arise in situations that require a different equilibrium angle, such as walking on varied terrain or using shoes with a different heel height.

**Fig 1:**
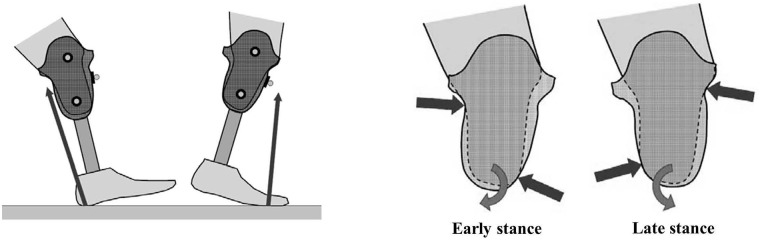
Drawings showing the loading extremes for level walking. The ground reaction force is behind the distal end of the socket in early stance phase and in front of it in late stance phase (left). The location of the applied ground reaction force causes a change in direction of the distal end sagittal torque from clockwise to counter-clockwise (right). The areas of highest pressure between the residual limb and the socket are shown with arrows. For downhill walking, the “early stance” torque is expected to be increased and prolonged. For uphill walking, the “late stance” torque is expected to be increased and prolonged. Movements of the residual limb within the socket are exaggerated to illustrate the effects of early and late stance torques.

Among the numerous adverse health outcomes associated with insufficient terrain adaptation, increased torque applied to the distal end of the prosthetic socket is perhaps the most detrimental. Studies of prosthetic alignment have found that when the equilibrium angle of the prosthetic ankle is not optimized for the walking surface, increased torque applied to the distal end of the socket can increase pressure within the socket, increase discomfort and skin breakdown on the residual limb [4–7], increase walking instability [6,8], and trigger compensatory muscle activity at the knee joint [5], increasing both energy consumption and perceived exertion. During normal, level-ground walking, the torque applied to the distal end of the socket typically transitions from a negative, external flexion moment to a positive, external extension moment according to the center of pressure (CoP) and orientation of the ground reaction force (GRF) vector (Fig 1, left). In the absence of terrain adaptation, abnormal loads applied to the limb during downhill walking can significantly increase early-stance socket reaction moments, resulting in excessive pressure on the anterior-distal and posterior-proximal regions of residual limb (Fig 1, right). Likewise, abnormal loads applied to the limb during uphill walking can significantly increase late-stance socket reaction moments, corresponding to excessive pressure on the anterior-proximal and posterior-distal regions of the residual limb (Fig 1, right). Over time, pain associated with increased socket pressures may limit activities of daily living for many individuals who have a sensate residual limb and who use non-adaptable ankle-foot prostheses.

To address this limitation, several hydraulic ankle-foot prostheses have been designed to improve non-level walking, such as the Echelon ankle-foot prosthesis (Endolite; Miamisburg, Ohio), which permits up to 9° of damped ankle motion (6° plantarflexion and 3° dorsiflexion relative to neutral). Compared to a rigid or non-adaptable ankle, studies show that individuals who walk with the Echelon ankle on level terrain exhibit a reduction in the magnitude of posteriorly-directed CoP displacements as well as a reduction in the variability of CoP velocity during single-support phase [9,10]. Individuals also translate more quickly over their prosthesis during early stance phase and increase their self-selected walking speed, suggesting that the hydraulic ankle unit may enable a smoother transfer of body weight onto the prosthesis during level walking [9,10].

To date, however, only two studies have evaluated the performance of a commercially-available hydraulic ankle during sloped walking tasks. Shortly after its commercial release, Portnoy et al. [11] conducted a study of the Echelon ankle in which the internal stresses in the residual limb were approximated using three pressure sensors attached to the distal end of the tibia. Although an oversimplification of residual-limb geometry and tissue composition were required to model internal stresses, results from this study indicated that the hydraulic ankle may be effective at reducing peak internal stresses in the residual limb during uphill and downhill walking (magnitude of walking slopes not reported). Likewise, Sedki et al. [12] reported that after a four-week take-home trial, a small group of transtibial and transfemoral amputees rated the Echelon ankle higher in a patient satisfaction survey compared to their standard, non-adaptable ankle. However, aggregate scoring across six domains of function (i.e., ambulation, transferring, utility, well-being, prosthesis satisfaction, and gait satisfaction) provided limited insight into the correspondence of these results to walking on inclines and declines.

**Fig 2:**
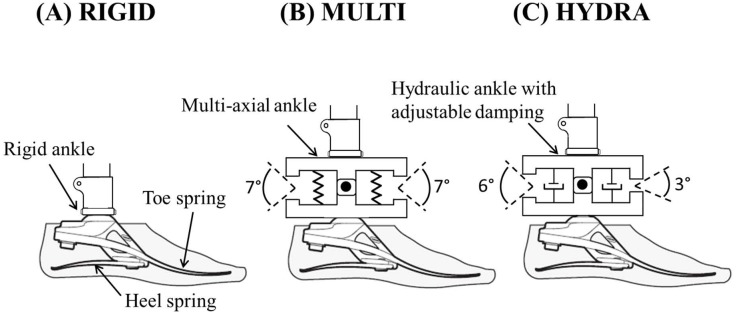
Schematic drawings of the: (A) Endolite Esprit ankle-foot system (RIGID) with a dynamic-response foot (i.e., carbon toe and heel spring as shown in manufacturer’s specifications) and rigid ankle, (B) Endolite Epirus ankle-foot system (MULTI) with a dynamic-response foot and multi-axial ankle, and (C) Endolite Echelon ankle-foot system (HYDRA) with a dynamic-response foot and hydraulic ankle with adjustable damping.

Beyond these preliminary studies, little evidence exists to support claims that a hydraulic ankle improves adaptation to sloped terrain. Therefore, the primary aim of this study was to investigate the effect of a passive, hydraulic ankle (HYDRA) on the socket reaction moments of individuals with unilateral transtibial amputation during a range of sloped walking tasks. To minimize the confounding effects of foot plate design and ankle range-of-motion (ROM), we compared the hydraulic Echelon ankle to two non-hydraulic prostheses built on the same modular platform with identical carbon toe and heel springs (Fig 2): the Endolite Esprit (dynamic-response foot, rigid ankle; RIGID) and the Endolite Epirus (dynamic-response foot, non-adaptable, multi-axial ankle; MULTI). Comparisons between the HDYRA and RIGID systems were designed to isolate the functional contribution of the hydraulic unit from the mechanics of the dynamic-response foot. Comparisons between the HYDRA and MULTI systems were designed to investigate the effect of hydraulic (i.e., damping-based impendence) versus elastic (i.e., stiffness-based impendence) ankle ROM. Given the potential for the Echelon ankle to adapt to sloped terrain, we hypothesized that individuals using the hydraulic ankle would experience less pronounced peaks in socket reaction moments compared to non-adaptable ankles when walking on sloped surfaces. Furthermore, we hypothesized that when walking with the hydraulic ankle, loads applied to the prosthesis would be similar to those at an equivalent location within the lower leg (shank) of a speed-matched, able-bodied control group, resulting in an increase in socket comfort and a decrease in perceived exertion while walking on sloped surfaces.

## Methods

### Recruitment

This study was approved by the Minneapolis VA Health Care System's Institutional Review Board: 4244-A Biomechanical Evaluation of Adaptable Ankle-Foot Prostheses. Subjects with unilateral transtibial amputation were recruited to participate in this study according to the following inclusion criteria:1) 18 to 70 years old, 2) free from neurological/musculoskeletal disorders, 3) able to walk without an assistive device, 4) able to walk on a treadmill without undue fatigue or health risks, 5) Medicare Functional Classification Level K2 or K3 prosthesis users (determined by a certified prosthetist), both of whom would encounter sloped terrain while walking in the community, 6) one or more years of experience with a definitive prosthesis, and 7) free from pressure sores on the residual limb. In addition, able-bodied, control subjects were recruited such that the age distribution of the control group matched that of the amputee group. Prior to enrollment, all subjects signed a consent form approved by the Minneapolis VA Health Care System’s Institutional Review Board.

### Experimental protocol

In total, subjects completed five study visits, with data collection sessions (visits 3–5) separated by two weeks. During the first visit, all subjects practiced walking on a split-belt treadmill (Bertec Corporation; Columbus, Ohio) equipped with an overhead safety harness, lateral hand rails, and an emergency stop button. Subjects in the amputee group wore their usual prosthesis during this practice session and were instructed to walk at their normal, self-selected walking speed. Although subjects were permitted to use the hand rails to assist with balance early in the practice session, they were encouraged to use the hand rails as little as possible as they grew accustomed to the treadmill. Subjects practiced walking on five slopes (10° decline, 5° decline, level, 5° incline, and 10° incline) for a total of 10–15 minutes. Self-selected walking speeds (i.e., treadmill belt speeds) were recorded for each slope. Finally, a certified prosthetist duplicated the subjects’ existing prosthetic socket in order to create a dedicated socket for the study.

On a second visit, subjects in the amputee group returned to the Minneapolis VA Health Care System to be fitted with their duplicate socket, a rigid pylon, and one of the following Endolite ankle-foot prostheses: Esprit (component weight = 317 g), Epirus (component weight = 402 g), or Echelon (component weight = 688 g). Subjects received prostheses that were appropriate for their weight and activity level and the order in which they received these prostheses was randomized. Subjects were instructed to wear the same shoes for each fitting. A certified prosthetist optimally aligned all components based on the manufacturer’s recommendation and their clinical experience. Subjects were then given two weeks to accommodate to their new prosthesis. During this time, subjects were instructed to wear the prosthesis as much as possible and to engage in normal activities of daily living.

Subjects then returned to the Minneapolis VA Health Care System for data collection. During this visit, subjects walked on the split-belt treadmill (harnessed and hands free) for several minutes at a constant, self-selected speed (i.e., the slowest belt speed recorded during the extreme sloped conditions of the practice session). Data were collected for each of five sloped walking conditions: 10° decline, 5° decline, level, 5° incline, and 10° incline. As with foot type, slope order was randomized by having subjects or an uninformed study staff member select a random sequence of numbers (i.e., 1, 2, 3), which corresponded to a particular sequence of slopes (i.e., level, 5°, 10°). Following each walking condition, subjects were asked to rate their socket comfort and walking effort using a Socket Comfort Score and a Rating of Perceived Exertion. The Socket Comfort Score is an 11-point scale that ranges from 0 (worst comfort imaginable) to 10 (greatest comfort imaginable) and is highly associated with socket fit [13]. The Rating of Perceived Exertion is a modified Borg scale [14] that allows subjects to rate their level of effort from 0 (no exertion) to 10 (maximum possible exertion). Consistent with previous studies, a change of 2 or more points was considered a clinically significant difference in socket comfort and walking effort [14,15].

This protocol was repeated for the remaining two ankle-foot prostheses. At the end of the study, subjects in the amputee group ranked the three components (best to worst) and provided feedback about walking on sloped surfaces. Subjects in the control group followed a similar protocol, with the exception that all control subjects walked at the mean, self-selected walking speed of the amputee group.

### Data collection

**Fig 3:**
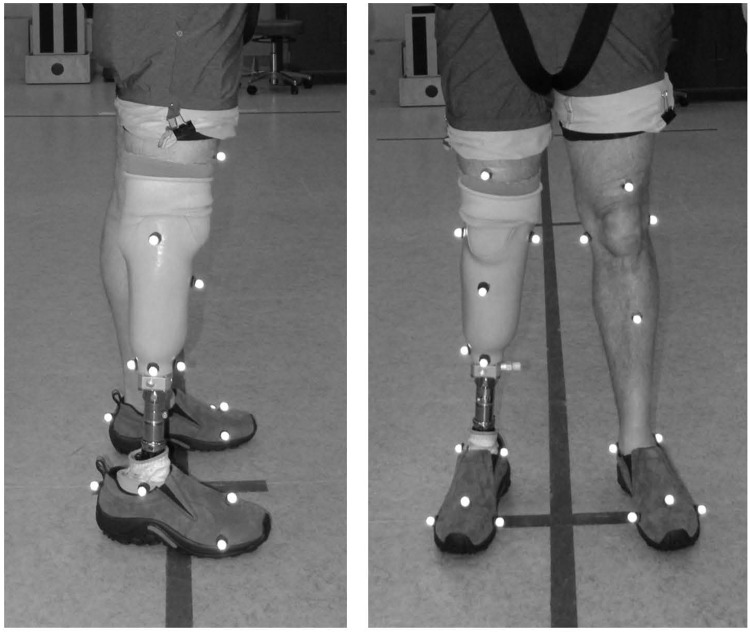
Markers placed on the distal end of the socket were used to define a socket coordinate system.

Motion data were tracked using an 8-camera Oqus 100 motion analysis system (Qualisys Motion Capture Systems; Gothenburg, Sweden) and were sampled at 120 Hz. Spherical, retro-reflective markers were placed on subjects according to a modified Helen Hayes full-body marker set [16]. Anatomical landmarks used during subsequent data analysis included the dorsum of each foot slightly proximal to the first, second, and fifth metatarsal heads, the posterior calcaneus at the height of the second metatarsal marker, the medial and lateral malleoli, and the medial and lateral femoral epicondyles. Non-collinear markers were also placed on the shank segments. The distal end of the prosthetic socket was tracked with markers placed on the anterior, lateral, and posterior end of the residuum (Fig 3). Force data were collected with an instrumented Bertec treadmill and sampled at 120 Hz. Given the inherent uncertainty of CoP data at low force levels, a force threshold of 500 N was applied to GRF data. Three, 10-second trials (capturing approximately 7 to 10 strides) were collected for each experimental condition.

### Data analysis

Marker data were processed using Qualisys Track Manager (QTM 2.8) then exported along with force data into MATLAB^®^ (R2014b, Mathworks, Inc.; Natick, Massachusetts) for further analysis. Marker data were filtered using an eighth-order, low-pass, bi-directional Butterworth filter with an effective cut-off frequency of 6 Hz. Force data were filtered using a fourth-order, low-pass, bi-directional Butterworth filter with an effective cut-off frequency of 12 Hz. A custom MATLAB^®^ script was written to calculate socket reaction moments at the distal end of the socket according to a three-dimensional quasi-static approach [17,18]. Socket reaction moments were transformed into the coordinate system of the socket, which was defined using the three markers placed at the anterior, posterior, and lateral end of the residuum (Fig 3). The origin of the socket coordinate system was defined as the midpoint between the anterior and posterior markers. From the sagittal-plane socket reaction moment, two variables were extracted for further analysis: minimum moment and maximum moment during single-limb support. To average across subjects, socket reaction moments were normalized to body mass (Nm/kg).

In order to compare the amputee and control groups, the residual-limb length for each amputee subject was calculated. The length of the residual limb was defined as the distance between the knee center and the origin of the socket coordinate system (i.e., midpoint between the anterior and posterior socket markers). This length was then normalized by the length of the amputee’s intact shank segment, defined as the distance between the knee center and ankle center (Table 1). Given that control subjects had intact limbs and did not use a prosthetic socket, equivalent socket reaction moments were calculated for each control subject at a shank location equal to the mean, normalized residual-limb length of the amputee group. Equivalent socket reaction moments were then transformed into a shank-based coordinate system. at a speed of 0.65 m/s to match the average walking speed of the amputee group.

**Table 1:** Demographic data of subjects with unilateral transtibial amputation.

Subject	Gender	Age (years)	Height (cm)	Mass (kg)	Residuum Length*	Self-Selected Treadmill Walking Speed (m/s)	K Level
1	M	50	182	115	0.42	0.60	K3
2	M	63	191	117	0.52	0.50	K3
3	M	58	178	82	0.37	0.60	K3
4	M	65	175	92	0.38	0.60	K3
5	M	64	175	81	0.56	0.90	K3
6	M	62	175	77	0.51	0.60	K3
7	M	40	180	88	0.58	0.80	K3
Mean (SD)		57 (9)	179 (6)	93 (16)	0.48 (0.08)	0.66 (0.14)	

* Normalized residual limb length = ratio of residual limb length (i.e., knee center to distal end) to the intact shank length (i.e., knee center to ankle center)

The orientation of the shank segment was also analyzed to determine the extent to which socket reaction moments were influenced by shank kinematics. For this analysis, the shank segment’s coordinate system was oriented such that its long axis was directed from the ankle joint center to the knee joint center. The sagittal-plane projection of the long-axis of the shank was then used to define a shank to vertical angle (SVA) according to Owen [19]. This measurement describes the degree of incline (negative angle) or recline (positive angle) of the shank relative to the force of gravity. Two variables were extracted from the SVA profiles for further analysis: minimum angle and maximum angle during single-limb support.

### Statistical analysis

All statistical analyses were performed using SPSS 19 for Windows (SPSS Inc.; Chicago, Illinois). Data points extracted from kinematic and kinetic profiles were averaged across all trials within each experimental condition for each subject. A repeated measures analysis of variance (ANOVA) was performed on data from the amputee group with two within-group factors (walking slope and ankle type). A Greenhouse-Geisser correction was applied when sphericity was violated. In the event of a significant main effect (p<0.05), post-hoc Bonferroni multiple comparisons were used to assess statistical significance.

To compare Echelon data to the control group, a two-way mixed ANOVA was performed with one between-group factor (HYDRA vs control) and one within-group factor (walking slope). Diagnostic testing for this analysis included Mauchly’s test of sphericity and Levene’s test of homogeneity of variance. A Greenhouse-Geisser correction was applied when sphericity was violated. In the event of a significant main effect (p<0.05), post-hoc Bonferroni multiple comparisons were used to assess statistical significance.

## Results

### Subject demographics

Data were collected from seven male subjects with unilateral transtibial amputation (Table 1). Amputation etiology varied across subjects and included vascular disease (n = 2), trauma (n = 3), cancer (n = 1), and a bone infection (n = 1). The mean age, mass, and height of the amputee group was 57 ± 9 years (ranging from 40–65 years), 93 ± 16 kg, and 179 ± 6 cm, respectively. The mean self-selected walking speed of the amputee group was 0.66 ± 0.14 m/s (ranging from 0.5–0.9 m/s). Control data were also collected from seven, able-bodied, male subjects. The control group was both age- and speed-matched to the amputee group. The mean age, body mass, and height of the control group was 57 ± 8 years (ranging from 43–67 years), 95 ± 14 kg, and 180 ± 4 cm, respectively. For each sloped condition, subjects in the control group were instructed to walk.

### Socket reaction moment

**Fig 4:**
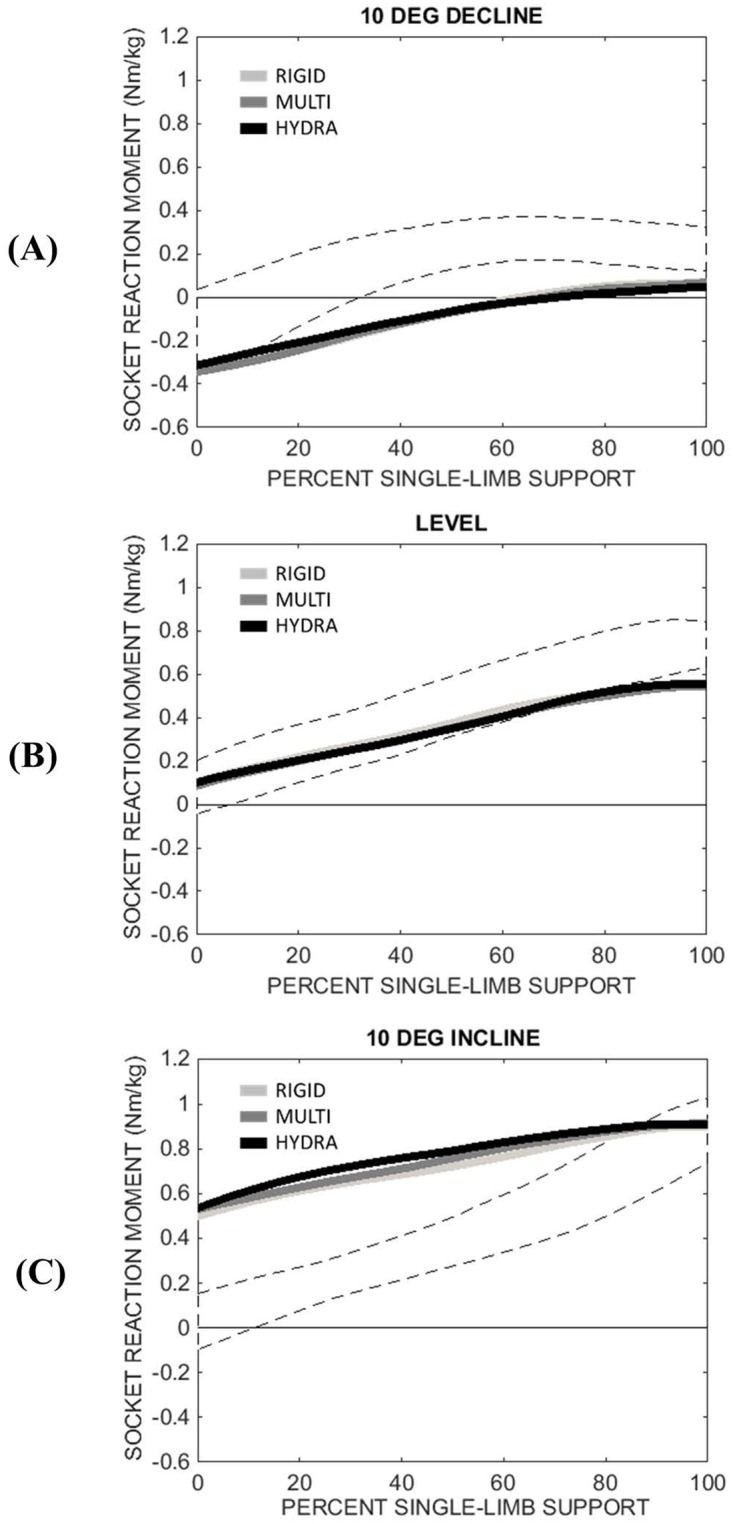
Mean socket reaction moments of subjects with transtibial amputation (n = 7) during single-limb support for the following sloped walking conditions: (A) 10° decline, (B) level, and (C) 10° incline. Dashed band corresponds to the mean reaction moment (± 1 standard deviation) of an able-bodied control group (n = 7) at a shank location equal to the mean, normalized residual-limb length of the amputee group. External extension moments are defined as positive.

**Fig 5:**
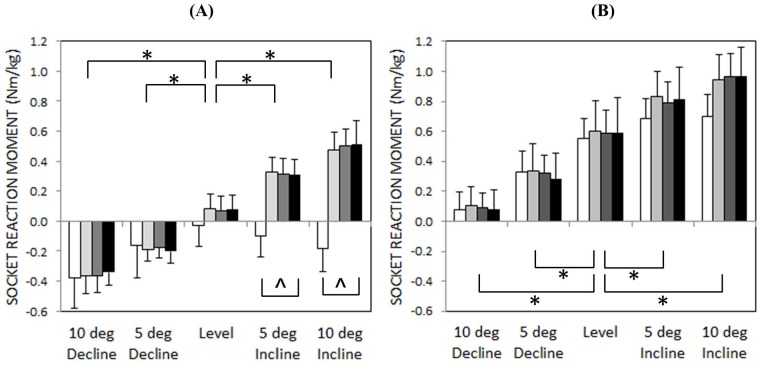
(A) Minimum and (B) maximum socket reaction moments (+ 1 standard deviation) during single-limb support (control = white, RIGID = light gray, MULTI = gray, HYDRA = black). External extension moments are defined as positive. An asterisk (*) indicates a statistically significant within-group difference (p<0.05) between level the sloped walking condition across all prosthetic feet. A carrot (^) indicates a statistically significant between-group difference (HYDRA vs control; p<0.05) within each sloped walking condition.

Sagittal-plane socket reaction moments calculated during single-limb support are shown in Fig 4. External extension moments are defined as positive. Standard deviation bands from the control group are shown for comparison. These bands correspond to a reaction moment across the shank segment at a level equal to the mean, normalized residual-limb length of the amputee group (48 ± 8% of the intact shank length). During level walking, moment data were similar between the control group and the amputee group. During sloped walking, however, socket reaction moments for the amputee group significantly increased/decreased for the 10° incline/decline conditions. Fig 5, which summarizes minimum and maximum socket reaction moments across all slopes, demonstrates this same trend. Statistical analysis revealed a significant main effect of walking slope on both minimum (F(1.571,9.427) = 189, *p*<0.001) and maximum (F(4,24 = 202.3, *p*<0.001) socket reaction moments for the amputee group, while ankle type did not significantly affect these peaks (minimum: F(2,12) = 0.021, *p* = 0.979; maximum F(2,12) = 0.128, *p* = 0.881).

By comparison, reaction moments of the control group were less affected by walking slope, particularly during early single-limb support when the minimum reaction moment remained negative across all sloped conditions (Figs 4 and 5). This trend resulted in a statistically significant interaction term between group (HYDRA vs control) and walking slope (*p*<0.001) and a statistically significant main effect of group for the minimum socket reaction moment during single-limb support (F(1,12) = 13.948, *p* = 0.003). Post-hoc analyses revealed that these differences were statistically significant for both the 5° incline (*p*<0.001) and 10° incline (*p*<0.001) conditions.

### Shank to vertical angle

**Fig 6:**
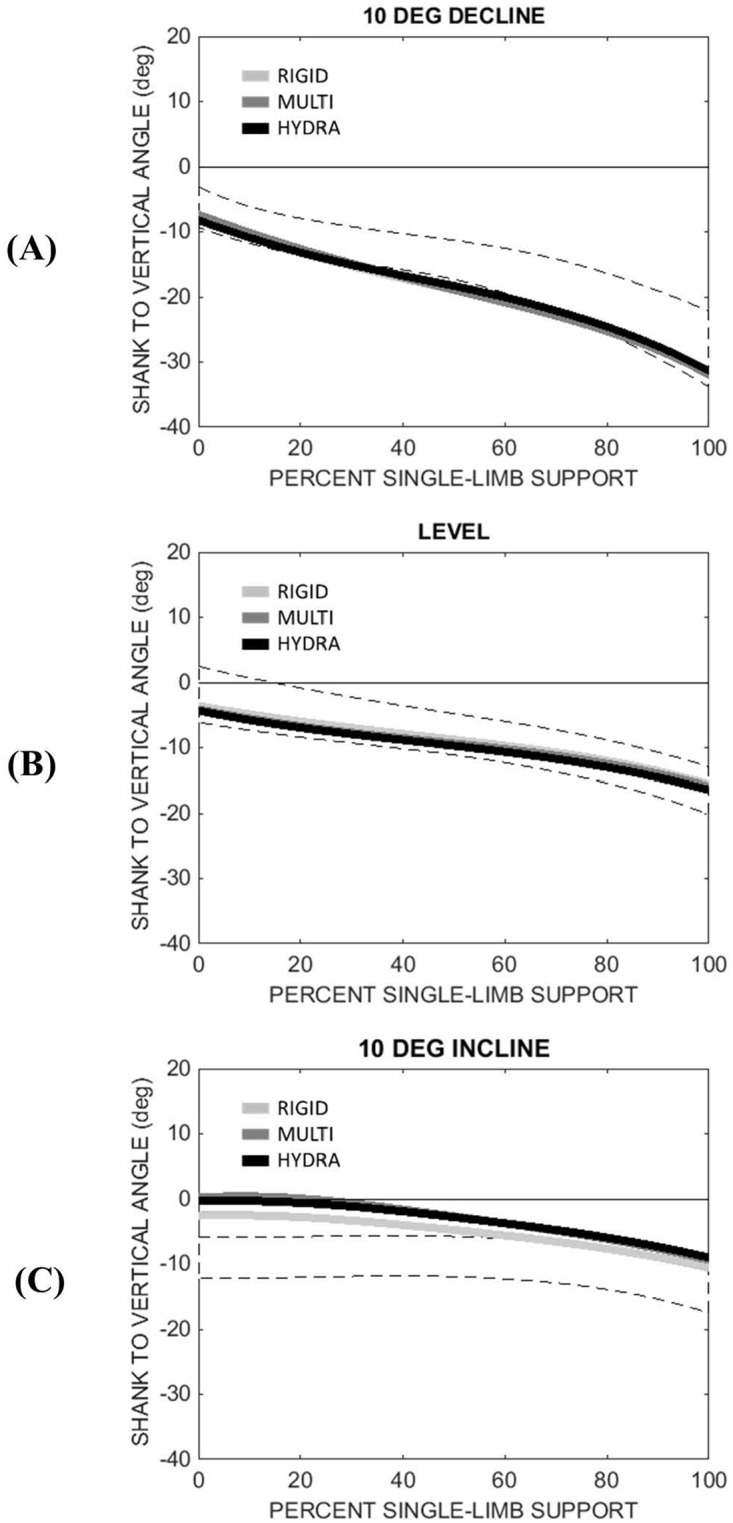
Mean Shank to Vertical Angle (SVA) of subjects with transtibial amputation (n = 7) during single-limb support for the following sloped walking conditions: (A) 10° decline, (B) level, and (C) 10° incline. Dashed band corresponds to the mean SVA (± 1 standard deviation) of an able-bodied control group (n = 7). Positive angles correspond to a reclined shank posture (i.e., the posture that would be expected at initial contact).

Fig 6 summarizes the SVA calculated during single-limb support. Positive angles correspond to a reclined shank posture (i.e., the posture that would be expected at initial contact). As seen in Fig 6, both control and amputee subjects maintained an inclined SVA for most of single-limb support regardless of walking slope. Furthermore, compared to level and inclined slopes, both groups exhibited an SVA curve that was more inclined while walking on a declined surface.

**Fig 7:**
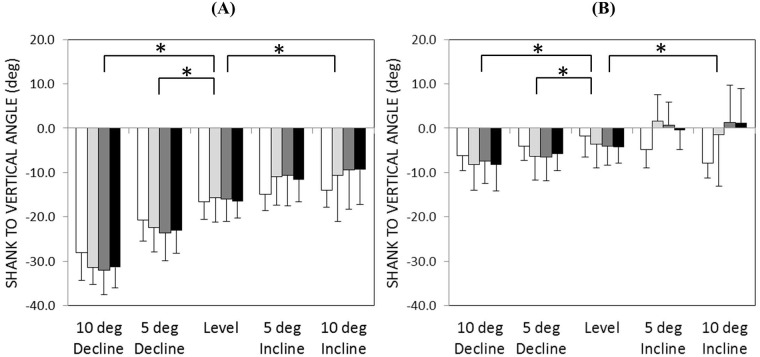
(A) Minimum and (B) maximum SVA (+ 1 standard deviation) during single-limb support (control = white, RIGID = light gray, MULTI = gray, HYDRA = black). Positive angles correspond to a reclined shank posture (i.e., the posture that would be expected at initial contact). An asterisk (*) indicates a statistically significant within-group difference (p<0.05) between level and the sloped walking condition across all prosthetic feet.

The most notable difference in SVA between the control group and amputee group can be seen during the first 40% of single-limb support. While walking on an inclined surface, the SVA for the amputee group was slightly reclined (i.e., positive) during early single-limb support, whereas it was slightly inclined (i.e., negative) for the control group (Fig 7). Statistical analyses revealed a significant interaction term between group (HYDRA vs control) and slope for maximum SVA (*p* = 0.001). However, the main effect of group was not statistically significant (F(1,12) = 0.483, *p* = 0.104).

With regard to the amputee group, a statistically significant main effect of walking slope was observed for both the minimum SVA (F(1.753,10.519) = 75.385, *p*<0.001) and maximum SVA (F(1.179,7.074) = 10.029, *p* = 0.014). Ankle type did not significantly affect either of these peaks (minimum: F(2,12) = 0.02, *p* = 0.980; maximum F(2,12) = 0.076, *p* = 0.927).

### Socket comfort

**Fig 8:**
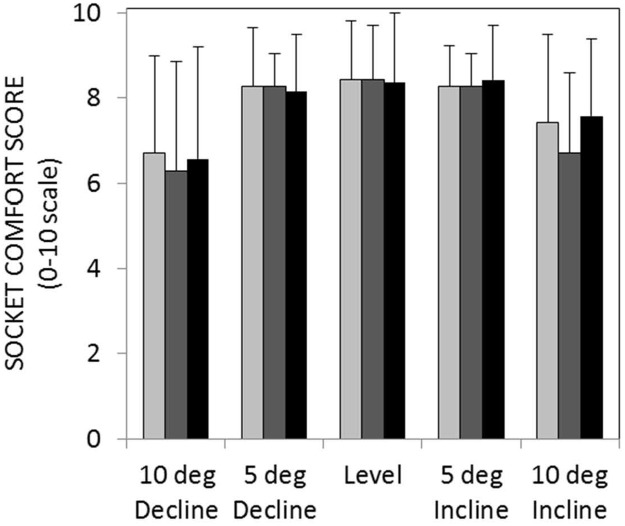
Mean socket comfort score (+ 1 standard deviation) across sloped walking conditions (RIGID = light gray, MULTI = gray, HYDRA = black). This 11-point scale ranges from 0 (worst comfort imaginable) to 10 (greatest comfort imaginable).

As expected, amputee subjects rated their socket most comfortable while walking on a level surface and least comfortable while walking on 10° of incline or decline (Fig 8). In fact, statistical analyses revealed a significant main effect of slope on self-reported ratings of socket comfort across all prosthetic ankle types (F(1.396,8.374) = 5.578; *p* = 0.037). In contrast, statistical analyses did not reveal a significant main effect of ankle type on self-reported scores of socket comfort (F(2,12) = 0.044, *p* = 0.957).

### Perceived exertion

**Fig 9:**
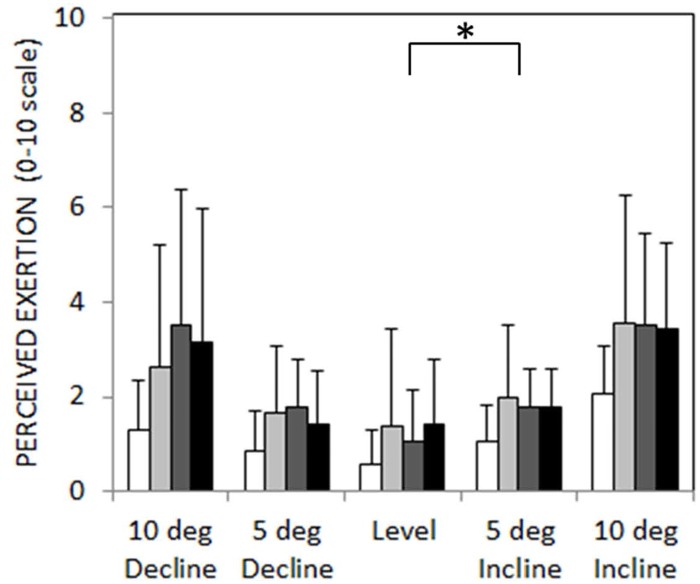
Mean rating of perceived exertion (+ 1 standard deviation) across sloped walking conditions (control = white, RIGID = light gray, MULTI = gray, HYDRA = black). This scale allows subjects to rate their level of effort from 0 (no exertion) to 10 (maximum possible exertion). An asterisk (*) indicates a statistically significant within-group difference (p<0.05) between level and the sloped walking condition across all prosthetic feet.

Corresponding to the trends observed in socket comfort, amputee subjects experienced the lowest level of exertion while walking on a level surface and the highest level of exertion while walking on 10° of incline or decline (Fig 9). Statistical analysis revealed a significant main effect of walking slope on perceived exertion scores across all prosthetic ankle types (F(1.732,10.39) = 6.32; *p* = 0.018). In contrast, perceived exertion was unaffected by ankle type (F(2,12) = 0.044; *p* = 0.957).

### Subjective feedback

Following data collection, subjects provided feedback about the ankle-foot prosthesis. At the conclusion of the study, five of the seven subjects also ranked their favorite and least favorite system based on its ability to facilitate uphill and downhill walking.

Of the five subjects who provided overall rankings, three indicated that the hydraulic ankle was their favorite (Table 1; S3, S6, S7); one indicated that they liked the rigid ankle just as well as the hydraulic ankle (S4), and one indicated that the multi-axial ankle was his favorite (S5). Of the three subjects who preferred the hydraulic ankle, one commented that he liked the ankle’s ROM and that he would like to use this ankle in his usual prosthesis (S6). Another commented that compared to the rigid ankle, the hydraulic ankle did not “wrench” his knee on sloped surfaces (S7). The subject who liked the hydraulic and rigid ankles equally thought that the hydraulic ankle would make it easier to climb ladders and get into and out of a boat (S4). Another subject commented that the hydraulic ankle would take the fear out of going down handicap ramps (S3).

Two subjects who criticized the hydraulic ankle felt that the device did not provide enough ROM when walking uphill (S5, S7). Furthermore, the subject who preferred the multi-axial ankle commented that the hydraulic ankle was not responsive to changes in walking speed and therefore, was not ideal for someone who is athletic (S5). This subject also described that he had to manually dorsiflex the hydraulic ankle when getting out of a chair, which was inconvenient.

## Discussion

Although several passive, hydraulic ankle-foot prostheses have been designed with the intent to improve sloped walking, data that quantify the extent to which these devices adapt to sloped terrain is currently lacking. This study compared the performance of a passive, hydraulic ankle-foot prosthesis to two related, non-hydraulic ankles based on their ability to minimize the socket reaction moments of individuals with transtibial amputation during a range of sloped walking tasks. As expected, we found that the design and alignment of all ankle-foot prostheses under investigation (RIGID, MULTI, and HYDRA) were sufficient for the demands of level walking. This assumption was based on the finding that during single-limb support, the calculated socket reaction moment profiles of the amputee group were similar to those of the able-bodied control group. Contrary to our hypothesis, however, the hydraulic ankle under investigation was unable to mimic the slope adaptation of the biological ankle-foot system, particularly when subjects walked on an inclined surface.

Previous studies of slope adaptation have shown that when encountering an inclined surface, able-bodied individuals re-orient the roll-over shape of their lower limb through kinematic adjustments of their ankle [1]. This re-orientation effectively changes the equilibrium angle of the ankle joint during stance phase, allowing the shank to pivot over the foot in a manner similar to that of level walking [20]. In this study, able-bodied individuals exhibited SVA profiles that were consistent across 0°, 5°, and 10° of incline (Figs 6 and 7) and peak reaction moments that were comparable between the level and inclined walking conditions (Figs 4 and 5). Together, these findings suggest an association between kinematic changes at the biological ankle joint and a relatively invariant loading pattern on the intact lower limb during slope ascent.

By comparison, loads transferred to the residual limb of individuals with transtibial amputation were significantly higher (compared to level) during slope ascent for all three ankle-foot mechanisms under investigation. This outcome was expected for both the rigid (RIGID) and multi-axial (MULTI) ankles, since these joints are designed to maintain a constant equilibrium angle. However, given the potential for the hydraulic ankle to adapt to slopes, we expected that the peak socket reaction moments of individuals wearing the hydraulic ankle would be similar between level and inclined walking. Instead, Fig 5 shows that the peak socket reaction moments calculated during inclined walking were significantly higher than those calculated during level walking as well as those calculated for able-bodied individuals walking on comparable inclines. This finding suggests that the ROM provided by the hydraulic ankle (i.e., 3° of viscoelastic dorsiflexion) was perhaps insufficient to meet the demands of sloped walking and that as a result, the performance of the hydraulic ankle was very similar to the non-adaptable rigid and multi-axial ankles.

Further evidence for this interpretation can be seen in Figs 6 and 7, which show that the SVAs measured during inclined walking were shifted toward a more reclined posture throughout single-limb stance compared to level walking. A reclined shank posture throughout stance phase may indicate a reduction in ankle dorsiflexion, which from a user’s perspective, would make it difficult to progress over the stance limb during slope ascent. Indeed, two subjects commented that the hydraulic ankle did not provide enough ROM for inclined walking and most considered their socket to be less comfortable when walking with the hydraulic ankle on an inclined versus level surface (Fig 8). It is interesting to note that the hydraulic ankle under investigation is not unique in its limited ankle dorsiflexion ROM. Compared to the Echelon ankle, which permits up to 3° of damped dorsiflexion (9° total ROM), the Kinterra ankle (Freedom Innovations; Irvine, California) permits only 2° of damped dorsiflexion (12° total ROM) and the MotionFoot (Fillauer; Chattanooga, Tennessee) permits up to 7° (50° total ROM). Given the primary role of the ankle joint in adapting to inclined surfaces, it may be beneficial for future versions of passive, hydraulic ankles to permit a larger range of ankle dorsiflexion in order to fully accommodate the demands of slope ascent. However, increasing the dorsiflexion range may also result in reduced performance for level walking, as it may lead to a reduced effective foot length on the prosthetic side and increased loading on the contralateral side [21,22]. To effectively mimic able-bodied walking on different slopes, the dorsiflexion stop angle may instead need to be controlled.

Similar to the inclined walking conditions investigated in this study, statistical analyses revealed that peak socket reaction moments calculated during declined walking were significantly different from those calculated during level walking and that there were no statistical differences between the hydraulic ankle and the two non-hydraulic ankles (Fig 4). However, unlike the inclined walking condition, post hoc comparisons between the hydraulic ankle and the able-bodied control group did not reveal a significant difference in the early single-limb stance socket reaction moment peak, indicating that the hydraulic ankle may have been better suited for the demands of declined walking. Perhaps this finding corresponds to the design of the hydraulic ankle, which provides a comparatively larger range of viscoelastic plantarflexion for declined walking. Alternatively, it is possible that kinematic adaptations at the knee joint may have augmented the apparent capacity of the hydraulic ankle to adapt to the declined walking surface. Indeed, previous studies have shown that the knee joint is crucial for adapting to declined walking surfaces [1]. Furthermore, while knee flexion was not explicitly investigated in this study, an increase in the inclined posture of the shank segment during declined walking suggests an increase in knee flexion (Figs 6 and 7), which may have played a larger role in managing peak socket reaction moments than the hydraulic ankle.

Despite limited evidence that that hydraulic ankle facilitated slope adaptation, three subjects in this study preferred the hydraulic ankle over a rigid or multi-axial ankle. Of course, it is difficult to attribute subjective preferences for the hydraulic ankle entirely to its performance on sloped walking surfaces. Similar to a previous study by Sedki and Moore [12], in which multiple domains of prosthesis function were evaluated (i.e., ambulation, transferring, utility, well-being, prosthesis satisfaction, and gait satisfaction), subjects may have biased their preference for the hydraulic ankle based on features that were not evaluated in this study. For example, when soliciting feedback from subjects at the end of the study, one user commented that it would be easier to climb a ladder or get into and out of a boat with the hydraulic ankle. Accordingly, although subject feedback provided additional context for the quantitative results of this study, subjective preferences for the hydraulic ankle were difficult to interpret given the small sample size and questionable correspondence of these results to walking on sloped surfaces. Furthermore, variation in amputation etiology could have differentially influenced perceptions of exertion and socket comfort.

It is also important to note that while the results of this study suggest that the hydraulic ankle under investigation was unable to mimic the slope adaptation of the biological ankle-foot system, these results correspond only to the single-limb support phase of gait while subjects walked on a treadmill. In order to investigate the contribution of the hydraulic ankle during the entire stance phase, including initial and terminal double-limb support, it would first be necessary to overcome limitations in the equipment used to measure kinetic data. Specifically, ground reaction force data measured by the treadmill in this study were subjected to a force threshold of 500 N, below which force data were unreliable. Unfortunately, the threshold required to obtain accurate socket reaction moment calculations precluded the ability to investigate residual limb loading during initial and terminal double support phases. However, future investigations may find that passive, hydraulic ankle-foot prostheses provide benefit during these important phases of initial and terminal limb loading.

To better understand the effect that a hydraulic ankle may have on compensatory strategies of the lower limbs and trunk, future investigation should also include a full analysis of joint kinematics and kinetics. In this study, markers that were placed on the pelvis were unfortunately obscured by the safety harness and surrounding structures of the treadmill, which prevented the ability to measure prosthetic knee flexion. Instead, SVA profiles were calculated to better understand the relationship between socket reaction moments calculated at the distal end of the residuum and the global orientation of the shank during different slope and prosthetic foot conditions. While this analysis provided insight into the kinematic strategies used by amputees to manage sloped terrain, this analysis was limited in that it did not reveal a particular joint strategy nor did it take into account the potential movement between the residual limb and prosthetic socket. Instead, because the markers used in this analysis were placed on the prosthetic socket, the SVA profiles reported in Fig 6 may be slightly different than the shifting angle of the residual limb within the socket.

In conclusion, this study was unable to find significant differences in the torque at the distal end of the residual limb socket between an ankle-foot prosthesis with hydraulic ROM and other common ankle-foot prosthesis designs (rigid ankle, multiaxial ankle) during the single-limb support phase of sloped walking. Also, socket comfort and perceived exertion were not significantly different for any of the ankle-foot prostheses tested in this study. None of the ankle-foot prostheses tested were able to mimic the shank kinematics and kinetics of able-bodied persons during the single-limb support phase of sloped walking. Further work is needed to determine if more advanced designs (e.g. those with microprocessor control of hydraulic features, powered ankle-foot designs) will provide more biomimetic function to prosthesis users.

## Supporting information

S1 Dataset Data used in all analyses. Minimum and maximum socket reaction moments; minimum and maximum shank to vertical angles (SVA); socket comfort scores; perceived exertion scores.
(XLSX)

## Abbreviations

ANOVA: analysis of variance
CoP: center of pressure
GRF: ground reaction force
HYDRA: hydraulic ankle
MULTI: multi-axial ankle
RIGID: rigid ankle
ROM: range-of-motion
SVA: shank to vertical angle
